# Diseases of the Teeth in Relation to So-Called Reflex Maladies

**Published:** 1885-03

**Authors:** Dudley W. Buxton

**Affiliations:** Assistant to the Professor of Medicine in University College


### Diseases of the Teeth in Relation to So-Called Reflex Maladies.

BY DUDLEY W. BUXTON, M.D., B.S., M.R.C.P., ASSISTANT TO THE PROFESSOR OF MEDICINE IN UNIVERSITY COLLEGE.

It may be taken as accepted that dental lesions give rise to diseased conditions in two ways. They may by direct continuity of tissue originate congestion and inflammation of the gums, while the same process may extend to the jaws and contiguous soft structures.

In the other case the local condition although the starting point of the trouble is yet dwarfed by the severity of some remote area of disease, the connection of which with the teeth is only assumed. The convulsions so common at teething are, according to various authors, cut to the most diverse conditions. Thus while Trousseau believes they result from the pyrexial state of the blood, a recent writer* holds that they occur independently of any elevation of temperature. Eclampsia again, it has been sought to explain, by supposing the hyperæmia which is present in the mouth structure, pervades the whole vascular and controlled by the vasomotor nerves of the head and neck. Barrier, however, adopts another view, asserting that the whole nervous system becomes unduly excited, owing to the impression made upon it by the pain experienced in the gums.

It is interesting to notice that some writers, especially of the French school, refer the dentition diarrhœa to a similar origin, Bouchut going so far as to term it "nervous diarrhœa," thus associating it with that form of diarrhœa of which Shylock tells us, and which manifests itself also amongst the emotional of adults.

In summing up it must be said that the preponderance of evidence shows that teething is more a predisposing than an exciting cause of trouble to children. At the same time warning is often given by the state of the gums and their departure from the physiological standard. This should always be recognized as an indication of some morbid process at work, and should receive the earnest care of the medical man, as well as the dentist. A search should be instituted to ascertain what is at fault and steps taken to remedy the evil before it has gone too far, by the intervention of the nervous system. It is more particularly with this latter class of cases that we have to deal. Although we are at present not capable of defining exactly which does, or what does

*Lewis Smith, Diseases of Children.

not, constitute reflex or sympathetic disease, yet we may attempt a description, always providing that it aims rather at elucidating pathological enigmas than their solution. We must understand the terms of our problem before we venture upon an explanation of its mysteries. Let us then take the simplest case. We have a nerve center, which performs the function of regulating tissue trophic changes, we have emissary nervous strands which perform the behests, so to speak, of the controlling centre; we have afferent nervous strands which travel from peripheral terminations to the centre. It is these last which maintain the centre *en rapport* with the outside world.

Pathology can not at present tell us at what point the afferent impulses of nerves, peripherally stimulated, may promote simple healthy growth and maintain the balance between destruction and regeneration, and at what point nutrition becomes deranged, the results of which we are cognizant being on the one hand hypertrophy, on the other atrophy.

We know that peripheral irritation acting upon nerves which are in connection with centres exerting trophic influence will, if allowed to act long enough, produce marked structural changes in tissues. The muscles of the arm become increased alike in bulk and power when subjected to frequent calls for action, but should so severe a strain be imposed upon the trophic centres as they can not meet, the beam is kicked and a pathological atrophy replaces physiological growth. The trophic centres, however, preside it may be, over a very wide area. One centre will dominate through its branches regions seemingly unconnected and widely remote alike in place and function.

A close physiological connection was thought at one time to exist between the nerve supply of skin and adjacent structures, a connection which probably really obtains even when coarse anatomy fails to demonstrate the nerve channels. Now it would appear that when the control and trophic centre is profoundly affected, that lesions result in one or more areas, due of course, to impaired or perverted nutrition. These areas may appear absolutely out of physiological relation until the connection is sought and found in the nervous supply and associated trophic centres. An example will illustrate it:

A patient attending the ophthalmic clinic of a general hospital complained of *discomfort* about left conjunctiva. This was congested and minute vesicles were detected upon it. It was elicited that severe unilateral headache had become manifest. Examination of the scalp showed vesicles along the line of the supra-orbital nerve. It was a case of herpes from irritation of the supra-orbital nerve with impairment of the nutrition of the globe of the eye. This is an example of a nerve--the fifth pair--giving rise to lesions in areas absolutely distinct, namely, those of the scalp and those of the eye.

It is, then, mainly the fifth pair of nerves which directly or indirectly gives rise to those innumerable aches and pains with the causation of which folks saddle the teeth. A glance at the anatomy of this fifth pair of nerves will serve to clear away some of the difficulties in tracing to the teeth such diverse and apparently disconnected lesions. The two roots of the nerve--motor and sensory, supply motor fibres to the masticatory muscles, sensation to the face, fore part of the head, the eye, the ear, the nose, and the mouth.

The first junction or anastomosis occurs at the Casserian ganglion where fibres from the sympathetic branches from the carotid plexus join it. The nerve then gives off its three large branches, the first going to the orbit and becoming connected with another ganglion--the ophthalmic--in which is communication with the third or motor nerve of the globe muscles and again with the sympathetic. The second going to supply the face and the teeth of the superior maxilla as well as the mucous membrane lining the maxillary sinus, the mucous membrane of the lips, that of the nose and upper portion of the pharynx as well as the tonsils, uvula, and soft palate. In the spheno-maxillary ganglion, in reality a plexus and not a ganglion, it communicates again with the sympathetic. The third division supplies the teeth of the lower jaw, the gums, the salivary glands, and articulation of the jaw, it also gives sensation to the side of the head, the external ear supplying the external meatus, the lower lip, and lower part of the face. The greater part of the tongue also obtains common sensation from this division. In the Otic-ganglion it becomes connected with the sympathetic and with the facial and glosso-pharyngeal nerves. The last named nerve supplies filaments to the pharynx, tympanum, and Eustachian tube, while we need hardly add, the facial nerve is the motor nerve of the muscles of the face. It is thus rendered plain upon anatomical grounds that the possible sources of nerve communicated by overflow of central nervous explosive action or central trophic changes, are numerous. But it is by no means accurate to assert that all the possible channels of nervous intercommunication between the fifth pair and other nerves are exhausted in the above anatomical account. There exist closer and more subtile series of possible channels in the physiological continuity of parts and association in function.

How commonly is the aspect of the tongue taken as indicative of the condition present in the mucous membrane of the stomach, and yet how seldom is the physiological connection subsisting between the tongue and the stomach realized or duly appreciated. In the cycle of physiological function in which participate the various sections of the alimentary apparatus there are many vicissitudes, and it is undoubted that as one part becomes out of gear so do the others suffer. In this connection we have to call attention to the common errors into which the laity and even the medical practitioner so commonly fall in referring to the process by which the milk teeth egress, those many maladies which appear in the first few years of infantile existence. It seems to be inseparable from most of the more active evolutions which occur in the human frame, that profound nervous impressions are called into existence, impressions which break down the inhibitory resistances and so irradiate throughout the wildest areas of the nervous system. The girl at puberty becomes the subject of such a nervous catastrophe, and in this way does the teething child show signs of the most profound upset of its nervous centres.

The teething is one sign of a general cycle of evolution. The metabolism of the whole bodily structure is being worked, so to speak, under high pressure. A shock too trivial to elicit conciousness in the well-balanced nervous system of the adult gives rise in the infant to a wide-spread and profound derangement of function and even of development.

Dr. Eustace Smith* draws attention to the fact that the follicles which are found studded along the whole alimentary tract are in process of development, and progress *pari passu* with the teeth, and hence alimentation must take place under the most strained conditions. And further we are bound to remember that although dentition is *per se* a physiological process, yet it taxes the children's strength, and coupled with the other processes of evolution at this time in active progress it is fully competent to kick the balance from the mean of health to the declension of disease. "Cross teething" may be symptomatic of an exhausted nervous centre, but when the trouble is in the tooth or kindred structures, it plays back in turn upon the central nervous system giving rise to some departure from the physiological cerebro-spinal function.

In fine, children suffer from convulsions *during* teething, but more rarely are such convulsions elicited *by* the eruption of the teeth. This point is fully worked out in the classical work of Barthez and Rilliet. As is well known, the pressure of the erupting tooth upon the gum gives rise to more or less stomatitis, a malady which may confine itself to the mouth in the region of the teeth in default, or which may spread far and wide. The nervous system is, as we pointed out above, peculiarly sensitive and impressionable, and it happens that the pyrexia caused by this stomatitis seriously disorders the child's powers of digestion, Thus, again, the vomiting, diarrhœa et cœt, ensue upon teething. and react upon the structures concerned in teething.

When morbid conditions are present, and teething has ceased to be a physiological process, we meet with some of those maladies, which form more particularly the subject of this paper. To stomatitis and gastro-intestinal catarrh we have referred above, aphthæ, vomiting, and diarrhœa, complications occurring usually in warm weather, need not detain us; nor would it be profitable to dwell upon the pulmonary and bronchial troubles which replace them when winter succeeds to summer. The whole body being as it were on the eve of disease, that organ or system feels the brunt of it, which casually becomes exposed to some exciting causes. —*British Journal of Dental Science.*

